# Breast cancer related lymphedema and shoulder mobility following radiotherapy

**DOI:** 10.1007/s00066-025-02482-0

**Published:** 2025-10-22

**Authors:** Tamara Jarm, Nikola Besic, Romi Cencelj Arnez, Jasna But-Hadzic, Ivica Ratosa

**Affiliations:** 1 Faculty of Medicine, University of Ljubljana, Ljubljana, Slovenia; 2 Division of Surgery, Institute of Oncology Ljubljana, Ljubljana, Slovenia; 3 Division of Radiotherapy, Institute of Oncology Ljubljana, Zaloska cesta 2, SI-1000 Ljubljana, Slovenia

**Keywords:** Breast cancer, Lymphedema, Reduced shoulder mobility, Axillary lateral thoracic artery

## Abstract

**Purpose:**

Lymphedema of the arm and reduced shoulder mobility are common complications of breast cancer treatment. We aim to establish whether the radiation dose received by the area of the axillary lateral thoracic artery vessel juncture (ALTJ) and the shoulder joint—affect the development of the mentioned side effects.

**Methods:**

In this retrospective study, 298 patients with early breast cancer treated surgically and with adjuvant radiation therapy, were included. Clinical data from the prospective database were used. Physiotherapists evaluated lymphedema and shoulder mobility at diagnosis, 6 and 12 months afterwards. The ALTJ, humeral head, and humeral head with a safety margin were delineated on a CT scan, and irradiation parameters were obtained from dose-volume histograms.

**Results:**

Multivariate analysis confirmed a correlation between higher mean (Dmean) and near-minimum (D98) radiation doses received by ALTJ and the incidence of lymphedema 12 months post-diagnosis (p = 0.016 and p = 0.002, respectively). No significant association was found between the radiation dose to the humeral head and reduced mobility.

**Conclusion:**

In our cohort of patients, irradiation of the ALTJ region is associated with the occurrence of clinically-assessed lymphedema, while irradiation of the humeral head is not linked to limited mobility of the shoulder after breast cancer treatment.

**Supplementary Information:**

The online version of this article (10.1007/s00066-025-02482-0) contains supplementary material, which is available to authorized users.

## Introduction

Breast cancer is the most common cancer affecting women as well as most diagnosed cancer worldwide, with over 2.3 million diagnoses yearly, and its prevalence is continuing to rise due to Western lifestyle factors [1]. Breast cancer treatment can lead to multiple side effects and long-term complications, impacting the patients’ psychophysical abilities and quality of life.

Lymphedema is a significant long-term complication, defined as regional swelling of the limb caused by injury to the lymphatic circulation. Lymphedema of the arm will develop in 10–40% of all breast cancer patients, 90% in the first year after treatment, but lifetime risk persists [2, 3]. The condition can be asymptomatic at first but progresses over time, leading to heaviness, pain, swelling, numbness, or tingling of the arm. During its development lymphatic fluid accumulates in the interstitial space, connective tissue proliferates and fat tissue hypertrophies, which thickens the skin and decreases its elasticity. Lymphedema can be diagnosed clinically by measuring the circumference of the arm above and below the elbow and comparing it to the measurements of the opposite arm or just based on symptoms. The treatment of lymphedema is challenging, most commonly including manual lymphatic drainage for 4–6 weeks to minimize the arm volume, followed by regular usage of elastic compression gloves, continuous lymphatic drainage, and physical activity to maintain the effect [2, 4, 5].

The risk factors for the development of lymphedema of the arm include the number of dissected lymph nodes, axillary dissection, radiotherapy of regional lymph nodes, and high body mass index [2–5]. The predicted probability of breast cancer-associated lymphedema with more conservative surgical techniques, such as sentinel lymph node biopsy, is 6 to 10% [2]. The effect of radiotherapy on the development of lymphedema is likely due to the development of fibrosis, which compresses the lymphatic vessels [4, 5]. Lately, a few studies have researched whether irradiation of axillary lateral thoracic vessel juncture (ALTJ), an area between the humeral head and the inferior-most contour of axillary vessels, increases the risk of lymphedema. ALTJ could be a control area for lymphatic drainage of the upper limb or a critical area for the regeneration of the lymphatic collateral vessels to the neighbouring lymphatic nodes, but its true importance is yet to be determined [6].

Reduced shoulder mobility is another possible breast cancer treatment complication, affecting everyday movements and commonly accompanied by pain and decreased muscle strength [7, 8]. Risk factors include the extent of the lymph node surgery and radiotherapy as with lymphedema, so the impact on irradiation of the humeral head must be considered [9].

The aim of this study is to evaluate whether the radiation dose received by the area of the ALTJ and the shoulder joint affect the development of the lymphedema of the arm and/or reduced shoulder mobility in patients following breast cancer treatment.

## Materials and methods

This study was approved by the institutional review board (ERID-KSOPR-0047/2023) and National Medical Ethics Committee (study approval number 0120-314/2023/3).

## Patient selection

**Fig. 1:**
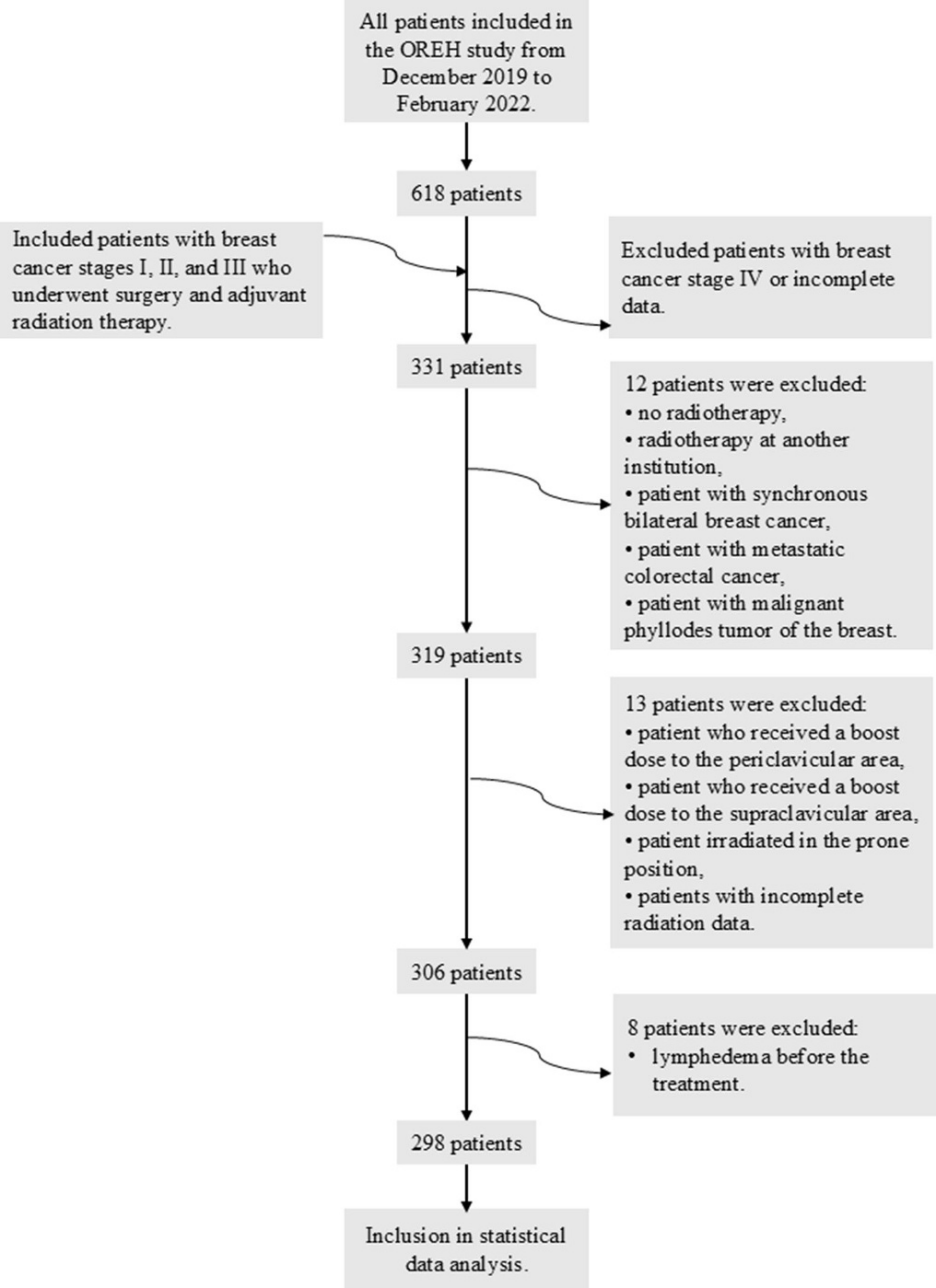
Patient cohort selection

Women with early-stage (I, II, III) breast cancer who received local treatment (surgery and adjuvant radiotherapy) with or without systemic treatment and participated in prior prospective study on early integrated and vocational rehabilitation (OREH study) from 2019 to 2022, were included [10, 11]. The whole inclusion and exclusion process is shown in Fig. 1.

## Data collection

The clinical data for the patient were taken from available medical documents. Patients’ symptoms and signs of lymphedema (tightness, tingling, heaviness, or swelling of the arm) and upper limb issues (restricted mobility, pain) were documented using patient-reported outcome measures (PROMs: OREH study questionnaires and standardized European Organisation for Research and Treatment of Cancer (EORTC) questionnaires QLQ-C30 and QLQ-BR23), which were completed by patients at diagnosis and 6 and 12 months later. Physiotherapists measured lymphedema and shoulder mobility at similar intervals. Lymphedema was evaluated by measuring arm circumferences 15 cm above and below the olecranon. A circumference difference of ≥ 2 cm between arms indicated lymphedema, according to previous literature [12]. Physiotherapists assessed upper arm range of motion (ROM) using goniometers to quantify joint movement. A discrepancy in ROM of more than 20 degrees between the upper limbs has been defined as restricted mobility.

## Definition of organs at risk and radiotherapy planning

**Fig. 2:**
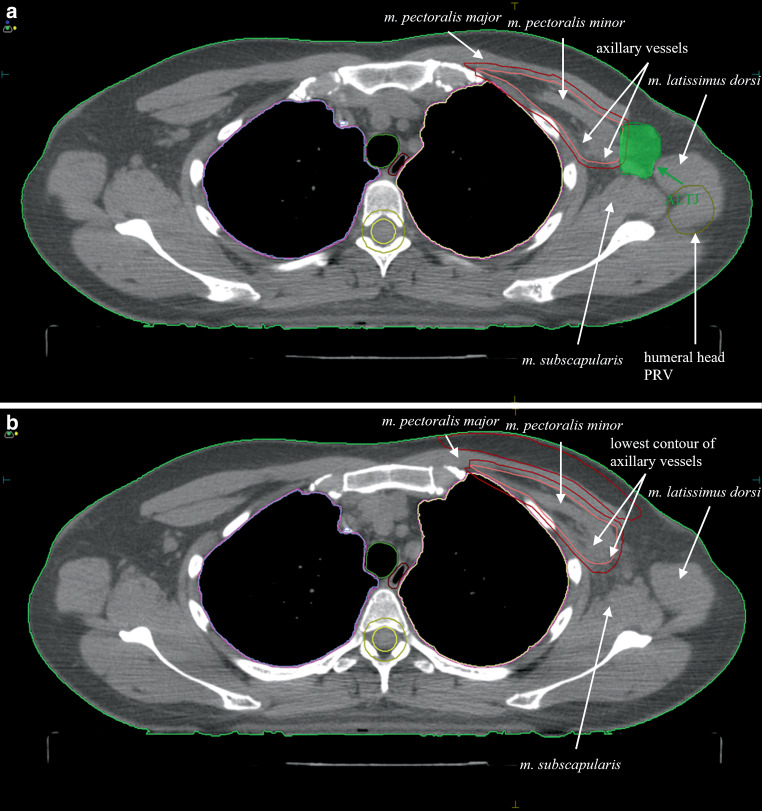
Delineation of the Axillary-Lateral Thoracic Vessel Juncture (ALTJ)—highest and lowest slice. The anatomical boundaries of the ALTJ are marked: the lowest contour of the humeral head, the planning risk volume of the humeral head, the pectoralis major muscle, the pectoralis minor muscle, the latissimus dorsi muscle, the subscapularis muscle, and the lowest contour of the axillary vessels

Clinical target volumes (CTV) of the breast, chest wall and groups I‑IV lymph nodes (LN), and LN along the internal mammary artery were identified from planning computed tomography (CT) images according to European Society for Radiotherapy and Oncology consensus guidelines [13]. On planning CTs ALTJ, humeral head and humeral head planning organ at risk volume (PRV) were delineated and the following metrics identified from the dose-volume histograms: Dx—dose received by x% of the target volume (x = 2, 98), Dmean—mean absorbed dose within a target volume, Vx—volume receiving x Gy (x = 10, 20, 30, 45, and 50). The contouring of ALTJ was delineated by TJ and validated by IR and JBH, adhering to a model proposed by Chang et al., as illustrated in Fig. 2; [14]. Radiotherapy treatment planning was conducted using Monaco® (Elekta AB, Sweden) and Varian Eclipse® (Varian, Palo Alto, CA) software. Patients received either three-dimensional conformal radiation therapy (3D-CRT) or intensity modulated radiation therapy (IMRT) per institutional guidelines.

## Statistical analysis

Statistical analyses were performed using IBM® SPSS® software package, version 30.0. (Statistical Package for the Social Sciences Statistical Software; SPSS Inc., Armonk: NY, IBM Corporation). Basic statistical analyses included calculation of the median, minimum, and maximum values. The normality of variable distributions was tested using the Kolmogorov-Smirnov and Shapiro-Wilk tests. Due to the predominantly non-normal distribution of variables, the Kruskal-Wallis test was applied to compare mean values. The Pearson chi-square test was used to assess the relationship between two categorical variables. Both univariate and multivariate analyses were performed. Descriptive statistics were used to summarize the characteristics of the study population. Variables with a *p*-value ≤ 0.10 from the univariate analysis were included in the final multivariate model. Statistical significance was defined for all differences with a *p*-value ≤ 0.05 (two-tailed test).

## Results

A total of 298 patients were included in the statistical analysis, their characteristics are listed in Supplementary Files Table 1. The median follow-up time was 34 months after diagnosis (range, 10–48 months) and 29 months after radiation therapy (range, 3–43 months). Dose-volume parameters of radiation treatment are shown in Supplementary Files Table 2.

## Association of dose-volume parameters and treatment methods with lymphedema occurrence

The cumulative incidence of physiotherapists-assessed lymphedema was 0 at diagnosis (0.0%), 101 at 6 months (35.2%) and 115 at 12 months after diagnosis (38.6%).

No significant difference in lymphedema occurrence was found depending on the extent of surgery (sentinel lymph node biopsy or axillary lymph node dissection) (*p* = 0.226). To assess the impact of radiation therapy on lymphedema patients were categorized in two groups: patients treated with breast or chest wall irradiation with or without LN regions I‑II irradiation and patients treated with breast, chest wall and LN regions I‑IV irradiation with or without the irradiation of LN along the internal mammary artery. Lymphedema occurred in 60 out of 177 patients (33.9%) in the first group, and in 55 out of 121 patients (45%) in the second group, showing a statistically significant difference (*p* = 0.044).

ALTJ volume overlap with target volumes (breast, chest wall, or regional LN regions) was identified in 153 (51.3%) radiation plans, with a slightly higher lymphedema incidence 12 months after diagnosis compared to patients without volume overlap in their radiation plans (43.1% vs. 33.8%), however, this difference was not statistically significant (*p* = 0.098).

According to the Kruskal-Wallis test, patients with higher body mass index (BMI), mastectomy, irradiation of I‑IV LN regions, and higher ALTJ D98 and Dmean values showed a statistically significantly higher risk developing lymphedema. The results are presented in Table 1. In the multivariate analysis, body mass index, extent of breast surgery, extent of irradiation of axillary regions, higher ALTJ D98 and higher ALTJ Dmean values were significantly associated with lymphedema, the results are shown in Table 2. In the group of patients with breast-conserving surgery 70 out of 140 patients (33.3%) developed lymphedema, while in the group of patients with mastectomy 44 out of 87 patients (50.6%) had lymphedema. In patients with lymphedema, the average ALTJ D98 was 14.4 Gy and ALTJ Dmean 28.3 Gy, while in those without lymphedema the average ALTJ D98 was 9.5 Gy and ALTJ Dmean 24.1 Gy.

**Table 1:** Univariate analysis of associations between different variables and the occurrence of lymphedema 12 months after diagnosis.

Variable	ALND (lymphedema 39/89)	SLNB (lymphedema 76/209)	Total (lymphedema 115/298)
Age (< 51 vs ≥ 51 years)	*p* = 0.865	*p* *=* *0.037*	*p* *=* *0.049*
BMI (< 25.9 vs ≥ 25.9 [kg/m2])	*p* *=* *0.008*	*p* = 0.363	*p* *=* *0.029*
Extent of breast surgery (breast-conserving surgery vs mastectomy)	*p* = 0.088	*p* = 0.073	*p* *=* *0.009*
Number of LN removed (< 2 vs ≥ 2, median value)	*p* = 0.064	*p* = 0.663	*p* = 0.368
Stage (I vs ≥ II)	*p* = 0.444	*p* = 0.096	*p* = 0.115
The irradiated regions of axillary LN (0-II vs I‑IV)	*p* = 0.374	*p* = 0.115	*p* *=* *0.044*
ALTJ D2 (< 34.2 vs ≥ 34.2, mean value, Gy)	*p* = 0.801	*p* = 0.409	*p* = 0.118
ALTJ D98 (< 11.4 vs ≥ 11.4, mean value, Gy)	*p* *=* *0.019*	*p* = 0.202	*p* *=* *0.016*
ALTJ Dmean (< 25.7 vs ≥ 25.7, mean value, Gy)	*p* = 0.234	*p* = 0.373	*p* *=* *0.042*
ALTJ V50 (< 1.1 vs ≥ 1.1, mean value, cm3)	*p* = 0.940	*p* = 0.079	*p* = 0.181
ALTJ V45 (< 3.3 vs ≥ 3.3, mean value, cm3)	*p* = 0.987	*p* *=* *0.011*	*p* = 0.055
ALTJ V30 (< 6.5 vs ≥ 6.5, mean value, cm3)	*p* = 0.178	*p* = 0.371	*p* = 0.051
ALTJ V20 (< 7.3 vs ≥ 7.3, mean value, cm3)	*p* = 0.244	*p* = 0.528	*p* = 0.086
ALTJ V10 (< 8.3 vs ≥ 8.3, mean value, cm3)	*p* = 0.317	*p* = 0.690	*p* = 0.172
ALTJ overlap with target volumes (no vs yes)	*p* = 0.206	*p* = 0.353	*p* = 0.098

ALND axillary lymph node dissection, SLNB sentinel lymph node biopsy, BMI body mass index, LN lymph nodes, ALTJ axillary lateral thoracic vessel juncture, Dx dose received by x% of the target volume, Dmean mean absorbed dose within a target volume, Vx volume receiving x Gy

**Table 2:** Multivariant analysis of variables associated with the occurrence of lymphedema 12 months after diagnosis.

Variable	HR, CI, *p*-value
Age (< 51 vs ≥ 51 years)	0.96 (0.93–0.99), *p* = 0.014
BMI (< 25.9 vs ≥ 25.9 [kg/m2])	1.09 (1.03–1.15), *p* *=* *0.005*
Extent of breast surgery (breast-conserving surgery vs mastectomy)	1.27 (0.64–2.55), *p* = 0.495
Chemotherapy (no vs yes)	1.12 (0.60–2.10), *p* = 0.720
The irradiated regions of axillary LN (0-II vs I‑IV)	1.29 (0.46–3.66), *p* = 0.630
Fractionation (conventional fractionation vs hypofractionation)	0.82 (0.31–2.14), *p* = 0.680
Radiation technique (3D-CRT vs IMRT)	1.24 (0.58–2.65), *p* = 0.583
ALTJ overlap with target volumes (no vs yes)	1.36 (0.43–4.26), *p* = 0.597
ALTJ D98 (< 11.4 vs ≥ 11.4, mean value, Gy)	1.05 (1.02–1.08), *p* *=* *0.002*
ALTJ Dmean (< 25.7 vs ≥ 25.7, mean value, Gy)	0.95 (0.90–0.99), *p* *=* *0.019*
ALTJ V45 (< 3.3 vs ≥ 3.3, mean value, cm3)	1.01 (0.92–1.11), *p* = 0.952
ALTJ V30 (< 6.5 vs ≥ 6.5, mean value, cm3)	0.93 (0.71–1.22), *p* = 0.593
ALTJ V20 (< 7.3 vs ≥ 7.3, mean value, cm3)	1.12 (0.87–1.44), *p* = 0.393

HR hazard ratio, CI 95% confidence interval, BMI body mass index, LN lymph nodes, ALTJ axillary lateral thoracic vessel juncture, Dx dose received by x% of the target volume, Dmean mean absorbed dose within a target volume, Vx volume receiving x Gy; 3D-CRT three-dimensional conformal radiation therapy, IMRT intensity-modulated radiation therapy

## Association of dose-volume parameters with restricted mobility of the affected upper limb

At 12 months after diagnosis restricted mobility of the affected upper limb was present in 106 patients (34.6%). Kruskal-Wallis test showed significant association between multiple dose-volume parameters and restricted mobility (Supplementary files Table 3), but multivariate analysis found no significant link (Table 3).

**Table 3:** Multivariate analysis of the dose-volume parameters associated with restricted mobility of the ipsilateral upper limb 12 month after diagnosis.

Variable	HR, CI, *p*-value
Humerus D_2_	1.06 (1.0–1.12), *p* = 0.057
Humerus D_98_	1.07 (0.81–1.41), *p* = 0.655
Humerus D_mean_	1.00 (0.84–1.18), *p* = 0.980
Humerus V_45_	1.39 (0.41–4.72), *p* = 0.596
Humerus V_30_	0.94 (0.67–1.30), *p* = 0.690
Humerus V_20_	1.21 (0.89–1.65), *p* = 0.227
Humerus V_10_	0.88 (0.75–1.03), *p* = 0.115
Humerus PRV D_2_	0.98 (0.94–1.02), *p* = 0.244
Humerus PRV D_mean_	1.08 (0.84–1.40), *p* = 0.549
Humerus PRV V_45_	0.97 (0.83–1.12), *p* = 0.640
Humerus PRV V_30_	0.94 (0.83–1.07), *p* = 0.353
Humerus PRV V_20_	0.99 (0.88–1.12), *p* = 0.896
Humerus PRV V_10_	1.03 (0.96–1.10), *p* = 0.437

HR hazard ratio, CI 95% confidence interval, Humerus humeral head, Humerus PRV humeral head planning organ at risk volume, Dx dose received by x% of the target volume, Dmean mean absorbed dose within a target volume, Vx volume receiving x Gy

## Patient-reported outcome measures

At 6 and 12 months after diagnosis, 39/296 (13.2%) and 15/250 (6.0%) of patients reported a swollen arm or hand, while 121/296 (40.9%) and 6/160 (3.4%) reported difficulty raising or moving the arm sideways, and 114/297 (38.3%) and 5/166 (3.0%) reported any pain in the arm or shoulder.

## Discussion

The study explored the association between treatment techniques, radiation dose-volume parameters and the occurrence of lymphedema and restricted upper limb mobility in breast cancer patients.

The cumulative lymphedema incidence was 35.2% at 6 months and 38.6% at 12 months after diagnosis, which is consistent with the literature [2–5]. A significantly higher risk of lymphedema was observed in patients receiving radiation to LN regions I‑IV. Overlap between ALTJ and target volumes was found in 51.3% of cases, with a slightly increased but non-significant lymphedema risk. Efforts to define an anatomical region whose irradiation would be linked to lymphedema risk have been inconclusive so far. The few studies researching the connection between the dose-volume parameters of the ALTJ region and lymphedema are summarized in Table 4. Our multivariate analysis confirmed a significant association between ALTJ D98 and ALTJ Dmean and the occurrence of lymphedema 12 months after diagnosis (*p* = 0.016 and *p* = 0.042). These results suggest that even minimal ALTJ irradiation may contribute to lymphedema development.

**Table 4:** Summary of previous research examining the relationship between dose-volume parameters of the ALTJ region and the occurrence of lymphedema [6, 14–16].

Author, research year	Gross et al., [6]	Park et al., [15]	Healy et al., [16]	Chang et al., [14]	Current study, Jarm et al., 2025
*N* of included patients	265	1345	378	1449	298
Research type	Retrospective	Retrospective	Retrospective	Retrospective	Retrospective
*Follow-up (months, median)*	*36*	*78.7*	*70*	*77.3*	*12*
Lymphedema diagnosis	Difference in arm circumference measured 15 cm above and 10 cm below the olecranon increased at consecutive visits (after treatment, if lymphedema signs/symptoms appeared, and at each follow-up)	Based on the Guidelines for the Diagnosis and Treatment of Lymphedema after Cancer Treatment by the Korean Lymphedema Society or through objective (volume measurement, bioimpedance, lymphoscintigraphy) and subjective (symptoms and signs) assessments	Difference in shoulder circumference between the ipsilateral and collateral limb was > 2.5 cm at any visit or ≥ 2 cm at ≥ 2 visits	Based on the Guidelines for the Diagnosis and Treatment of Lymphedema after Cancer Treatment by the Korean Lymphedema Society or through objective (volume measurement, bioimpedance, lymphoscintigraphy) and subjective (symptoms and signs) assessments	Difference in arm circumference measured 15 cm above and below the olecranon. A circumference difference of ≥ 2 cm between arms indicated lymphedema
Established relationship between ALTJ dose-volume parameters and lymphedema: yes or no	Yes	Yes	No.	Yes	Yes
Dose-volume parameters statistically significantly associated with lymphedema occurrence	ALTJ D_min_	All dose-volume parameters except Dmin: ALTJ D_max_, D_mean_, V_5_, V_10_, V_15_, V_20_, V_25_, V_30_, V_35_, V_40_, V_45_, V_50_	NA	All dose-volume parameters: ALTJ D_min_, D_max_, D_mean_, V_5_, V_10_, V_15_, V_20_, V_25_, V_30_, V_35_, V_40_, V_45_, V_50_	ALTJ Dmean and ALTJ D98

*N* number, ALTJ axillary-lateral thoracic vessel juncture, Dx dose received by x% of the target volume, Dmean mean absorbed dose within a target volume, Vx volume receiving x Gy, NA Not available

The first study examining the association between the ALTJ region and lymphedema was a 2019 study by Gross et al., which included 265 patients with stage II and III breast cancer treated with surgery and radiation. They confirmed an association between ALTJ radiation dose and the risk of lymphedema, with the most convincing variable being a Dmin < 36.8 Gy (3-year lymphedema incidence 5.7% vs 37.4%, *p* value < 0.001) [6]. A similar 2023 study by Healy et al., included 378 patients and found no association between ALTJ metrics and lymphedema [16].

In a 2023 study by Park et al., based on 1345 patients, a multivariable model was developed to predict the risk of lymphedema in breast cancer patients treated with radiation therapy. The highest three- and five-year incidence of lymphedema was found in patients with more than 10 LN removed and ALTJ V35 Gy > 39.9% (18.7% and 25%), while the incidence of lymphedema in patients with only one of these risk factors (5.9%) or without both risk factors (0.5% and 0.9%) was significantly lower [15]. A 2023 study by Chang et al., which included 1449 patients, examined whether ALTJ dose-volume parameters inclusion improved lymphedema risk prediction model. They found the lowest risk of lymphedema in patients with ≤ 6 LN removed and ALTJ V35 Gy ≤ 66%, with a 5-year incidence of 1.2% and the highest risk in patients with > 15 LN removed and ALTJ Dmax > 53 Gy, with a 5-year incidence of 71.4% [14].

Taking altogether, studies have shown that irradiation of lymph node areas [17], larger radiation volumes (including more lymphatic structures) [18, 19], and higher radiation dose to the lymph node area [6, 14, 15, 19] all increase the risk of lymphedema, most likely by causing more severe fibrosis and vascular damage, making lymphatic obstruction more likely [20]. The results whether ALTJ should be identified as an organ at risk (OAR) remain contradictory, although several studies support its role as a potential risk factor for lymphedema development. Dosimetric constraints for particular axillary structures have yet to be integrated into clinical practice, as additional prospective studies are required (e.g., investigating optimized radiotherapy planning and incorporating sparing techniques that consider lymphatic vessel anatomy), along with individualized imaging of the lymphatic system, given that lymphatic drainage in the arm may differ among patients [21]. The primary risk factors for lymphedema remain the extent of axillary LN surgery, the number of LN removed, adjuvant radiation, advanced stage and higher BMI [20, 22].

The cumulative incidence of restricted mobility of the ipsilateral upper limb 12 months after breast cancer diagnosis was 34.6%, aligning with literature. Multivariate analysis found no statistical association between humerus volume-dose parameters and lymphedema. To date, only one research, by Belaidi et al., investigated the humeral head as a potential OAR, analysing late adverse events in 159 patients undergoing breast cancer radiation. The average dose to the humeral head was 9.18 Gy and the maximum 24.41 Gy, but no statistical link with the occurrence of adverse events was found [23]. To validate data from retrospective research, humeral head and surrounding soft tissue should be included in future prospective dosimetric studies as a possible OAR.

Our study is the first in Slovenia to investigate whether the ALTJ’s dose-volume parameters influence the development of lymphedema, as well as the effect of humeral head irradiation on the incidence of limited mobility in the adjacent upper limb. To the best of our knowledge, there have been very few studies on this topic published. The strength of our study is precise ALTJ region delineation (with no inter-observership variability), a relatively large patient cohort and regular prospective follow-up intervals (6 and 12 months). Additionally, we analysed dose-volume parameters for both sentinel lymph node biopsy and axillary dissection. Our study’s limitations include the use of a single definition of lymphedema, measured by circumference difference of ≥ 2 cm. As definitions vary in the literature, our findings may not be directly comparable to those of other studies. However, a main limitation is the short 12 months follow-up, since late irradiation consequences like fibrosis can occur later. Furthermore, although we collected PROMs at regular intervals, we did not consider their association with radiation therapy. This publication only presents a subset of the data collected from a prospective institutional study. Additional datasets (particularly the association between PROMs and exposure to radiation therapy), beyond the scope of this research, are being analyzed and will be explored in future publications.

## Conclusions

In this study, we investigated the impact of radiation on common side effects after breast cancer treatment. We found that the near minimal and mean dose received by the ALTJ, near the target radiation volumes, is important for the occurrence of lymphedema 12 months after diagnosis. The association between the dose received by the humeral head and limited mobility of the adjacent shoulder joint was not found.

## Supplementary Information

Table A1 Patient, tumor and treatment characteristics of the patient cohort (*n* = 298).

Table A2 Dosimetric-volumetric parameters of irradiation of the ALTJ region, humeral head, and humeral head PRV.

Table A3 Univariate analysis of dose-volume parameters associated with restricted mobility of the ipsilateral shoulder joint 12 months after treatment.

## Data Availability

The datasets generated and/or analysed during the current study are available from the corresponding author upon reasonable request.

## References

[1] Arnold M, Morgan E, Rumgay H, Mafra A, Singh D, Laversanne M et al (2022) Current and future burden of breast cancer: global statistics for 2020 and 2040. Breast 66:15–23. 10.1016/j.breast.2022.08.010 36084384 10.1016/j.breast.2022.08.010 PMC9465273

[2] Rockson SG (2018) Lymphedema after breast cancer treatment. N Engl J Med 379(20):1937–1944. 10.1056/NEJMcp1803290 30428297 10.1056/NEJMcp1803290

[3] Taghian NR, Miller CL, Jammallo LS, O’Toole J, Skolny MN (2014) Lymphedema following breast cancer treatment and impact on quality of life: a review. Crit Rev Oncol Hematol 92(3):227–234. 10.1016/j.critrevonc.2014.06.004 25085806 10.1016/j.critrevonc.2014.06.004

[4] Gillespie TC, Sayegh HE, Brunelle CL, Daniell KM, Taghian AG (2018) Breast cancer-related lymphedema: risk factors, precautionary measures, and treatments. Gland Surg 7(4):379–403. 10.21037/gs.2017.11.04 30175055 10.21037/gs.2017.11.04 PMC6107585

[5] Tsai RJ, Dennis LK, Lynch CF, Snetselaar LG, Zamba GKD, Scott-Conner C (2018) Lymphedema following breast cancer: the importance of surgical methods and obesity. Front Womens Health. 10.15761/FWH.1000144 30555923 10.15761/FWH.1000144 PMC6293280

[6] Gross JP, Lynch CM, Flores AM, Jordan SW, Helenowski IB, Gopalakrishnan M, Cutright D, Donnelly ED, Strauss JB (2019) Determining the organ at risk for lymphedema after regional nodal irradiation in breast cancer. Int J Radiat Oncol Biol Phys 105(3):649–658. 10.1016/j.ijrobp.2019.06.2509 31260718 10.1016/j.ijrobp.2019.06.2509

[7] Lovelace DL, McDaniel LR, Golden D (2019) Long-term effects of breast cancer surgery, treatment, and survivor care. J Midwifery Womens Health 64(6):713–724. 10.1111/jmwh.13012 31322834 10.1111/jmwh.13012

[8] Zhang X, Wang C, Fan J, Murakami S, Xie H, Huo M (2024) The factors influencing shoulder mobility disorders in patients after radical breast cancer surgery: a cross-sectional study. Breast Care 19(1):43–48. 10.1159/000535063 38384491 10.1159/000535063 PMC10878701

[9] Leonardis JM, Lulic-Kuryllo T, Lipps DB (2022) The impact of local therapies for breast cancer on shoulder muscle health and function. Crit Rev Oncol Hematol 177:103759. 10.1016/j.critrevonc.2022.103759 35868499 10.1016/j.critrevonc.2022.103759 PMC9706536

[10] Kovacevic N, Žagar T, Homar V, Pelhan B, Sremec M, Rozman T, Besic N (2024) Benefits of early integrated and vocational rehabilitation in breast cancer on work ability, sick leave duration, and disability rates. Healthc 12(23):2433. 10.3390/healthcare12232433 10.3390/healthcare12232433 PMC11641150 39685055

[11] Auprih M, Zagar T, Kovacevic N, Smrdel ACS, Besic N, Homar V (2024) Impact of early integrated rehabilitation on fatigue in 600 patients with breast cancer—a prospective study. Radiol Oncol 58(2):243–257. 10.2478/raon-2024-0016 38452328 10.2478/raon-2024-0016 PMC11165971

[12] Arnold M, Morgan E, Rumgay H, Mafra A, Singh D, Laversanne M et al (2022) Current and future burden of breast cancer: global statistics for 2020 and 2040. Breast 66:15–23. 10.1016/j.breast.2022.08.010 36084384 10.1016/j.breast.2022.08.010 PMC9465273

[13] Kaidar-Person O, Vrou OB, Hol S, Arenas M, Aristei C, Bourgier C et al (2019) ESTRO ACROP consensus guideline for target volume delineation in the setting of postmastectomy radiation therapy after implant-based immediate reconstruction for early stage breast cancer. Radiother Oncol 137:159–166. 10.1016/j.radonc.2019.04.010 31108277 10.1016/j.radonc.2019.04.010

[14] Chang SJ, Ko H, Im HS, Kim SJ, Byun KH, Kim BY et al (2023) Incorporating axillary-lateral thoracic vessel juncture dosimetric variables improves model for predicting lymphedema in patients with breast cancer: A validation analysis. Clin Transl Radiat Oncol 41:100629. 10.1016/j.ctro.2023.100629 37131951 10.1016/j.ctro.2023.100629 PMC10149196

[15] Park YI, Chang JS, Ko H, Im SH, Kim JS, Byun HK et al (2023) Development and validation of a normal tissue complication probability model for lymphedema after radiation therapy in breast cancer. Int J Radiat Oncol Biol Phys 116(5):1218–1225. 10.1016/j.ijrobp.2023.01.056 36739918 10.1016/j.ijrobp.2023.01.056

[16] Healy E, Beyer S, Jhawar S, White JR, Bazan JG (2023) The axillary lateral vessel thoracic junction is not an organ at risk for breast cancer-related lymphedema. Int J Radiat Oncol Biol Phys 117(2):452–460. 10.1016/j.ijrobp.2023.04.003 37059233 10.1016/j.ijrobp.2023.04.003

[17] Byun HK, Chang JS, Im SH, Kirova YM, Arsene-Henry A, Choi SH et al (2021) Risk of Lymphedema following contemporary treatment for breast cancer: an analysis of 7617 consecutive patients from a multidisciplinary perspective. Ann Surg 274(1):170–178. 10.1097/SLA.0000000000003491 31348041 10.1097/SLA.0000000000003491

[18] Nassif T, Brunelle C, Gillespie T, Bernstein M, Bucci L, Naoum G et al (2020) Breast cancer-related lymphedema: a review of risk factors, radiation therapy contribution, and management strategies. Curr Breast Cancer Rep 12:305–316. 10.1007/s12609-020-00387-8

[19] Warren LE, Miller CL, Horick N, Skolny MN, Jammallo LS, Sadek BT et al (2014) The impact of radiation therapy on the risk of lymphedema after treatment for breast cancer: a prospective cohort study. Int J Radiat Oncol Biol Phys 88(3):565–571. 10.1016/j.ijrobp.2013.11.232 24411624 10.1016/j.ijrobp.2013.11.232 PMC3928974

[20] Shen A, Lu Q, Fu X, Wei X, Zhang L, Bian J, Qiang W, Pang D (2022) Risk factors of unilateral breast cancer-related lymphedema: an updated systematic review and meta-analysis of 84 cohort studies. Support Care Cancer 31(1):18. 10.1007/s00520-022-07508-2 36513801 10.1007/s00520-022-07508-2

[21] Boneti C, Korourian S, Bland K, Cox K, Adkins LL, Henry-Tillman RS et al (2008) Axillary reverse mapping: mapping and preserving arm lymphatics may be important in preventing lymphedema during sentinel lymph node biopsy. J Am Coll Surg 206(5):1038–1042. 10.1016/j.jamcollsurg.2007.12.022 18471751 10.1016/j.jamcollsurg.2007.12.022

[22] Ribeiro PACP, Koifman RJ, Bergmann A (2017) Incidence and risk factors of lymphedema after breast cancer treatment: 10 years of follow-up. Breast 36:67–73. 10.1016/j.breast.2017.09.006 28992556 10.1016/j.breast.2017.09.006

[23] Belaidi L, Loap P, Kirova Y (2022) Do we need to delineate the humeral head in breast cancer patients? Cancers 14(3):496. 10.3390/cancers14030496 35158764 10.3390/cancers14030496 PMC8833338

