# Supplementary material for: Breast cancer related lymphedema and shoulder mobility following radiotherapy
Source: Strahlenther Onkol. 2025 Oct 22;202(2):187–95. doi: 10.1007/s00066-025-02482-0 (PMC12872703; doi:10.1007/s00066-025-02482-0)
Supplement: Supplementary file 1 — Table A1 Patient, tumor and treatment characteristics of the patient cohort (n = 298). [file 66_2025_2482_MOESM1_ESM.docx]

| **Characteristics** | | |
| --- | --- | --- |
| Age, median ([IQR]) [years] |  | 51.1 (44–58) |
| BMI, median ([IQR]) [kg/m2] |  | 25.9 (22.3–28.4) |
| Stage, n (%) | Not defined | 5 (1.7) |
|  | I | 132 (44.3) |
|  | II | 112 (37.6) |
|  | III | 49 (16.4) |
| **Locoregional treatment** |  |  |
| Surgery, n (%) | No surgery | 1 (0.3) |
|  | Lumpectomy | 210 (70.5) |
|  | Mastectomy | 87 (29.2) |
| LN surgery, n (%) |  |  |
|  | Sentinel LN biopsy | 209 (70.1) |
|  | Axillary dissection | 89 (29.9) |
| N of positive LN, median ([IQR]) |  | 0 (0-36) |
| N of removed LN, (median ([IQR]) |  | 2 (0-42) |
| Radiation therapy, n (%) | Yes | 298 (100) |
| Area of radiation, n (%) | Breast/chest wall | 172 (57.7) |
|  | Breast/chest wall + regional LN | 126 (42.3) |
| LN radiation, n (%) | Without or levels I-II | 177 (59.4) |
|  | Levels I-IV + LN along the internal mammary artery | 121 (40.6) |
| Boost, N (%) | No | 164 (55) |
|  | Yes - SIB | 86 (28.9) |
|  | Yes - Electrons | 44 (14.8) |
|  | Yes - Photons | 4 (1.3) |
| Radiation technique, n (%) | 3D-CRT | 198 (66.4) |
|  | IMRT | 100 (33.6) |
| Total radiation dose, median ([IQR]) [Gy] |  | 44.6 (40.1–50) |
| N of fractions, median ([IQR]) |  | 19 (15–25) |
| Fractionation |  |  |
|  | Conventional fractionation (1.8-2 Gy per day) | 104 (34.9) |
|  | Hypofractionation (>2 Gy per day) | 194 (65.1) |
| **Systemic treatment** | | |
| Chemotherapy, n (%) |  |  |
|  | Yes | 160 (53.7) |
|  | No | 138 (46.3) |

**Table A1**. Patient, tumor and treatment characteristics of the patient cohort (n = 298). Legend: IQR – interquartile range, BMI – body mass index, N – number, LN – lymph nodes, SIB –simultaneous integrated boost, 3D-CRT – three-dimensional conformal radiation therapy, IMRT – intensity-modulated radiation therapy.
