# Supplementary material for: Breast cancer related lymphedema and shoulder mobility following radiotherapy
Source: Strahlenther Onkol. 2025 Oct 22;202(2):187–95. doi: 10.1007/s00066-025-02482-0 (PMC12872703; doi:10.1007/s00066-025-02482-0)
Supplement: Supplementary file 2 — Table A2 Dosimetric-volumetric parameters of irradiation of the ALTJ region, humeral head, and humeral head PRV. [file 66_2025_2482_MOESM2_ESM.docx]

| Variable | ALTJ | Humerus | Humerus PRV |
| --- | --- | --- | --- |
| D_2_, mean (range) [Gy] | 34.2 (0.1-58.1) | 19.4 (0-51.0) | 27 (0.1-58.0) |
| D_98_, mean (range) [Gy] | 11.4 (0-49.6) | 0.9 (0-14.5) | 0.5 (0-2.6) |
| D_mean_, mean (range) [Gy] | 25.7 (0-55.5) | 4.6 (0-34.0) | 5.4 (0-30.0) |
| V_50_, mean (range) [cm^3^] | 1.1 (0-15.7) | 0 (0-0) | 0.1 (0-5.7) |
| V_45_, mean (range) [cm^3^] | 3.3 (0-24.3) | 0.1 (0-3.4) | 1.3 (0-21.3) |
| V_30_, mean (range) [cm^3^] | 6.5 (0-29.3) | 1.7 (0-41.1) | 9.4 (0-60.2) |
| V_20_, mean (range) [cm^3^] | 7.3 (0-30.5) | 3.9 (0-62.8) | 15.9 (0-85.9) |
| V_10_, mean (range) [cm^3^] | 8.3 (0-30.5) | 8.0 (0-78.2) | 26.9 (0-131.6) |

**Table A2**. Dosimetric-volumetric parameters of irradiation of the ALTJ region, humeral head, and humeral head PRV. Legend: ALTJ – axillary lateral thoracic vessel juncture, Humerus – humeral head, Humerus PRV –humeral head planning organ at risk volume, Dx - dose received by x % of the target volume, Dmean - mean absorbed dose within a target volume, Vx - volume receiving x Gy.
