# Supplementary material for: Breast cancer related lymphedema and shoulder mobility following radiotherapy
Source: Strahlenther Onkol. 2025 Oct 22;202(2):187–95. doi: 10.1007/s00066-025-02482-0 (PMC12872703; doi:10.1007/s00066-025-02482-0)
Supplement: Supplementary file 3 — Table A3 Univariate analysis of dose-volume parameters associated with restricted mobility of the ipsilateral shoulder joint 12 months after treatment. [file 66_2025_2482_MOESM3_ESM.docx]

| Variable | ALND  (restricted mobility of the upper limb 44/89) | SLNB  (restricted mobility of the upper limb 57/209) | Total  (restricted mobility of the upper limb 101/298) |
| --- | --- | --- | --- |
| Humerus D_2_ | p = 0.879 | p = 0.558 | **p = 0.004** |
| Humerus D_98_ | p = 0.706 | p = 0.608 | **p = 0.044** |
| Humerus Dmean | p = 0.834 | p = 0.319 | **p = 0.002** |
| Humerus V_50_ | p = 1.000 | p = 0.102 | p = 0.164 |
| Humerus V_45_ | p = 0.988 | **p = 0.023** | **p = 0.011** |
| Humerus V_30_ | p = 0.970 | p = 0.689 | **p = 0.008** |
| Humerus V_20_ | p = 0.685 | p = 0.565 | **p = 0.003** |
| Humerus V_10_ | p = 0.944 | p = 0.782 | **p = 0.011** |
| Humerus PRV D_2_ | p = 0.670 | p = 0.751 | **p = 0.008** |
| Humerus PRV D_98_ | p = 0.681 | p = 0.586 | p = 0.108 |
| Humerus PRV Dmean | p = 0.909 | p = 0.465 | **p = 0.005** |
| Humerus PRV V_50_ | p = 0.254 | p = 0.667 | p = 0.721 |
| Humerus PRV V_45_ | p = 0.663 | p = 0.932 | **p = 0.032** |
| Humerus PRV V_30_ | p = 0.863 | p = 0.902 | **p = 0.013** |
| Humerus PRV V_20_ | p = 0.787 | p = 0.994 | **p = 0.009** |
| Humerus PRV V_10_ | p = 0.857 | p = 0.866 | **p = 0.024** |

**Table A3**. Univariate analysis of dose-volume parameters associated with restricted mobility of the ipsilateral shoulder joint 12 months after treatment. Legend: ALND – axillary lymph node dissection, SLNB – sentinel lymph node biopsy, Humerus – humeral head, Humerus PRV –humeral head planning organ at risk volume, Dx - dose received by x % of the target volume, Dmean - mean absorbed dose within a target volume, Vx - volume receiving x Gy.
